# H2A Histone Family Member X (H2AX) Is Upregulated in Ovarian Cancer and Demonstrates Utility as a Prognostic Biomarker in Terms of Overall Survival

**DOI:** 10.3390/jcm9092844

**Published:** 2020-09-02

**Authors:** Sayeh Saravi, Eriko Katsuta, Jeyarooban Jeyaneethi, Hasnat A. Amin, Matthias Kaspar, Kazuaki Takabe, George Pados, Fotios Drenos, Marcia Hall, Emmanouil Karteris

**Affiliations:** 1 Department of Life Sciences, Brunel University London, Uxbridge UB83PH, UK; sayeh.saravi@brunel.ac.uk (S.S.); jeyarooban.jeyaneethi@brunel.ac.uk (J.J.); Hasnat.Amin@brunel.ac.uk (H.A.A.); 1722302@brunel.ac.uk (M.K.); Fotios.drenos@brunel.ac.uk (F.D.); 2 Department of Surgical Oncology, Roswell Park Comprehensive Cancer Center, Buffalo, NY 14263, USA; Eriko.Katsuta@RoswellPark.org (E.K.); kazuaki.takabe@roswellpark.org (K.T.); 3 1st Department of OB-GYN, Aristotle University, 541 24 Thessaloniki, Greece; padosgyn@gmail.com; 4 Mount Vernon Cancer Centre, Middlesex, Northwood HA6 2RN, UK

**Keywords:** H2AX, ovarian cancer, biomarker, polymorphisms

## Abstract

*Background*: H2AX can be of prognostic value in breast cancer, since in advanced stage patients with high levels, there was an association with worse overall survival (OS). However, the clinical relevance of H2AX in ovarian cancer (OC) remains to be elucidated. *Methods*: OC *H2AX* expression studied using the TCGA/GTEX datasets. Subsequently, patients were classified as either high or low in terms of *H2AX* expression to compare OS and perform gene set enrichment. qRT-PCR validated *in-silico H2AX* findings followed by immunohistochemistry on a tissue microarray. The association between single nucleotide polymorphisms in the area of H2AX; prevalence and five-year OC survival was tested in samples from the UK Biobank. *Results*: *H2AX* was significantly overexpressed in OCs compared to normal tissues, with higher expression associated with better OS (*p* = 0.010). Gene Set Enrichment Analysis demonstrated gene sets involved in G2/M checkpoint, DNA repair mTORC1 signalling were enriched in the H2AX highly expressing OCs. Polymorphisms in the area around the gene were associated with both OC prevalence (rs72997349-C, *p* = 0.005) and worse OS (rs10790282-G, *p* = 0.011). Finally, we demonstrated that H2AX gene expression correlated with γ-H2AX staining in vitro. *Conclusions*: Our findings suggest that H2AX can be a novel prognostic biomarker for OC.

## 1. Introduction

An acquired or inherited deficiency in DNA repair pathways in humans can lead to an increased lifetime risk of cancer. DNA double strand break (DSBs) is the most lethal insult to the genome and if left unrepaired, can result in genomic instability and cell death [1]. Normal cells have efficient mechanisms for double strand DNA (dsDNA) repair, involving many mechanisms to monitor DNA, identify damage and stop the cell cycle until repair is complete. Nevertheless, some people inherently have mutations that affect DNA repair pathways; these people have higher risk of developing genomic instability and cancer.

Examples of inheritable mutations in DNA repair genes which are known to increase the lifetime risk of cancers include *BRCA1* and *BRCA2*. Certain mutations of these two well-characterized genes, result in a significant risk of breast and ovarian cancer in carriers, due to defective homologous recombination (HR), the principal mechanism for DSBs repair [2]. In HR, DNA from the matching chromosome is used as a template for repair. Resection of the 5′ ends of each broken DNA strand occurs to leave overhangs of 3′ single DNA strands which invades the partner chromosomal DNA for repair in various different ways. *BRCA1* and *BRCA2* gene products are critical components of the HR system. Although HR deficiency (HRD) was first identified in cells carrying *BRCA1/2* germline mutations, such changes to other genes involved in the HR process are also described and can occur sporadically via genetic and epigenetic inactivation. The term BRCAness applies to the phenotypic outcome associated with changes in these other HR related genes, as this is very similar to that seen in patients with *BRCA1/2* mutations [3].

The histone protein H2AX, one of the isoforms of the histone H2A family and distinguished by the presence of a short COOH terminal tail, is closely associated with dsDNA repair; it is phosphorylated to γ-H2AX by member of phosphatidyl inositol 3-kinase family in response to DSB [4,5]. Large quantities of γ-H2AX accumulate rapidly around a nascent DSB. The consolidation of γ-H2AX in the chromatin at the dsDNA break site serves as a focus for HR repair machinery. The application of γ-H2AX antibodies to the amplified γ-H2AX, results in discrete foci appearing at sites of ds breaks within the nucleus [4,5] and its use as a biomarker in cancer chemotherapy is emerging [6].

Mice lacking H2AX show greater sensitivity to radiation and are less efficient at DSB repair which in turn cause a high level of chromosomal abnormalities [7]. This suggests that H2AX may also have a role in maintaining genomic stability. Similarly, loss of one or both H2AX alleles in mice result in genomic instability and a high risk of tumour progression in a p53 null background [8,9], corroborating a role for H2AX as a caretaker of the genome [10].

On average 30% of the genome is affected by chromosome arm-level or focal deletions in human cancer [11,12,13]. The distal 11q arm harbours the main DNA damage repair (DDR) genes including *ATM*, *MRE11A*, *CHEK1* and *H2AX*. In this context, it might be interesting that loss of distal 11q arm is frequently detected in different cancers and result in tumour progression. In a considerable proportion of head and neck squamous cell carcinomas, Parikh et al. discovered partial deletions of the region on 11q23 containing *H2AX*, suggesting a possible contribution of H2AX in human cancers [14]. Furthermore, the study indicates that other tumours categorized by loss of the distal region of chromosome 11q need to be analysed for loss of DNA repair efficiency [10].

In this study we investigated in detail the expression of *H2AX* in OC including population sample and genotyping. We have provided a better insight into the protein expression using a tissue microarray and provided evidence of transcriptional changes depending on the BRCA status.

## 2. Materials and Methods

### 2.1. Cell Lines and Clinical Samples

PEO1 adherent cell line was derived from ovarian adenocarcinoma with a defect in the HR DNA DSB repair pathway. The PEO4 cells are adherent ovarian cancer from the same patient as the PEO1 with differentiated serous adenocarcinoma. SKOV3 are adherent and hypo-diploid cells, derived from human epithelial OC patient. MDAH-2774 cells are also adherent, derived from the patient with ovarian endometrioid adenocarcinoma. Ovarian cancer samples (stage III) were obtained from patients at the 1st Department of Obstetrics and Gynecology, ‘Papageorgiou’ General Hospital, Medical School, Aristotle University, Thessaloniki, Greece. Ethical permission was obtained locally and informed consent was given.

### 2.2. Cell Culture

The cell lines were regularly cultured in T75 cell flasks with a filter head (Thermo Fisher Scientific UK, Hemel Hempstead, UK), supplemented with 10% foetal bovine serum and 1% penicillin-streptomycin (Thermo Fisher Scientific UK, Hemel Hempstead, UK). Cells were incubated at 37 °C in a humidified atmosphere of 5% CO_2_ in air. Cells were sub-cultured at 80% confluency, by trypsinization with TE (TrypLE Express, Thermo Fisher Scientific UK, Hemel Hempstead, UK). Cell counts and viability were detected with “Countess™” (Invitrogen, Carlsbad, CA, USA) automated cell counter which utilises the trypan blue exclusion method.

### 2.3. DNA Damage Induction by H_2_O_2_ Exposure

Cells were seeded on poly-prep slides (Poly-l-lysine coated glass slide, Sigma-Aldrich, St. Louis, MO, USA) for 24-h before treatment. Two slides of each cell line were retained as a control (untreated); the remaining slides were exposed to H_2_O_2_ (0.5 mM/1-h). Following exposure, the cells were returned to the incubator with fresh medium, and then removed at 1-h and 24-h after exposure.

### 2.4. Immunofluorescence—γ-H2AX Assay

In brief, cells were washed in phosphate buffered saline (PBS) then fixed using 4% paraformaldehyde (PFA) in PBS for 15 min. In the next step, cells were permeabilized using 0.2% Triton-X solution (Sigma-Aldrich) in dH_2_O for 10 min at 4 °C. Then, non-specific sites on the cells were blocked using 100 μL 2.5% bovine serum albumin (BSA) blocking buffer for each slide. Slides were covered with parafilm and placed in a humidified dark box for one hour. Next, a total of 100 μL of diluted γ-H2AX antibody conjugated with Alexa Fluor (Merck Millipore, Burlington, MA, USA), at the relevant concentration (1:100) (following manufacturer’s instructions), was added to the slides. The slides were recovered with parafilm and placed in a humidified dark box for one hour. After that, the slides were washed 3 times for 5 min in TBST (Tris-buffered saline with Tween 20, PH7.5, Sigma-Aldrich) and then PBS. The slides were de-hydrated in gradient ethanol series for 5 min each time. Once the slides were air dry, 15 μL DAPI (Vector Laboratories, Burlingame, CA, USA) was added and each slide covered with a cover slip and sealed using clear nail varnish. The slides were analysed under a DM4000 microscope (Leica, Milton Keynes, UK).

### 2.5. RNA Isolation, cDNA Synthesis and Quantitative RT-PCR

RNA was extracted from tissue lysate using the RNeasy^®^ Mini Kit (Qiagen, Hilden, Germany). cDNA was synthesised from mRNA with cDNA reverse transcription Kits (Applied-Biosystems, Foster City, CA, USA), cDNA concentration was controlled using RNA concentrations defined by Nano-Drop 2000C (Thermo Fisher Scientific UK, Hemel Hempstead, UK). Relative expression of the genes of interest was measured by quantitative PCR (qPCR) on QPCR QuantStudio 7 Flex Real-Time PCR machine using SYBR^®^ Green PCR Master Mix (Applied-Biosystems). The following primers were used as target gene H2AX: forward 5′-CGGGCGTCTGTTCTAGTGTT-3′ and reverse: 5′-GGTGTAC ACGGCCCACTG-3′; and housekeeping gene: YWHAZ, forward: 5′-AGACGGAAGGTGCTG AGAAA-3′, and reverse: 5′-GAAGCATTGGGGATCAAGAA-3′.

### 2.6. Immunohistochemistry for Tissue Microarray

Paraffin-embedded ovarian tissue microarray slides each containing 100 cores (Supplementary Table S1) were purchased from US Biomax Inc. (Rockville, MD, USA). The slides were deparaffinised and rehydrated, followed by antigen retrieval. Blocking was carried out with 5% BSA in PBS, followed by 24-h incubation with primary rabbit monoclonal antibodies for H2AX (Millipore) (1:500 dilution). Following numerous washes with PBS, the slides were incubated with secondary antibody 1:200 in 1% rabbit serum (ZytoChem Plus HRP-DAB Kit, Zytomed Systems GmbH, Berlin, Germany) for 60 min. After washing with 0.025% Triton-X, to remove any unbound secondary antibody, streptavidin-HRP conjugate was added to the slides with the secondary antibody and incubated for 30 min in the humidity chamber. The slides were then washed and subjected to DAB staining, counterstained with haematoxylin and washed with 0.1% sodium bicarbonate. The slides were analysed for immunoreactivity of H2AX protein by light microscope (Zeiss, Jena, Germany). Results were measured by calculating the percentage of positive tumour cells as previously described [15].

### 2.7. Bioinformatic Analysis

The ovarian cancer cohort of TCGA was downloaded through cBioPortal [16] and UCSC Xena (https://xena.ucsc.edu/). This cohort comprises of 568, 17, and 8 tissues of primary cancer, recurrent cancer, and normal ovarian tissue with gene expression from microarray in TCGA. OC patients with primary tumours were divided into high and low expression groups by *H2AX* expression using median cut-off. Gene Set Enrichment Analysis (GSEA) was carried out comparing *H2AX* high and low groups using Hallmark gene sets, in software provided by the Broad Institute (http://software.broadinstitute.org/gsea/index.jsp).

### 2.8. Population Sample and Genotyping

The UK Biobank is a large population study of approximately 500,000 individuals. These were recruited between 2006 and 2011 from 22 UK Biobank assessment centres throughout England, Wales and Scotland. The age range of the participants at the time of enrolment in the study was from 40 to 69 years, with a mean age of 56.5 years old. Females represent 54.4% of the sample [17]. Existing health records have been linked with the rest of the participants data. These include information on deaths, cancer registration and hospital inpatient episodes (http://biobank.ctsu.ox.ac.uk/crystal/crystal/docs/DataLinkageProcess.pdf). For this project we used the ICD10 definition of ovarian cancer under code C56 with 1232 cases. To determine the five-year survival, we used the dates of diagnosis of the incident cases with five-year follow up data and information from the death register for the next five years. 488,377 individuals have been genotyped for up to 812,428 variants using the UKB Axiom array (438,427 participants) or the UK BiLEVE Axiom array (49,950 participants). These variants were imputed using the 1000 Genomes phase 3 dataset as a reference [18]. Variants that did not pass standard quality control checks [18], those with a minor allele frequency of <0.01, or imputed variants with an INFO score of <0.8, were excluded from the analysis. All existing polymorphisms passing our QC filters and situated on chromosome 11 between positions 118,944,675 and 118,986,118, in total 108 genetic variants, were extracted from the genotyping data for use in this project. Sample genotyping quality control metrics were provided by the UK Biobank. Samples were excluded from the analysis if they were outliers for missingness and/or PC-corrected heterozygosity and/or if they had any sex chromosome aneuploidies as well as if the genetically inferred sex differed from the reported sex. Samples which did not have a genetically determined White British ancestry were also excluded. A list of related individuals was provided by the UK Biobank and one individual from each related pair was excluded at random. The use of the UK Biobank data was covered under project 44,556.

### 2.9. Statistical Analysis

Changes observed in experiments were assessed for statistical significance using the Student’s t-test and ANOVA (Analysis of Variance) test. The survival differences were analyzed using Kaplan-Meier curves with log-rank test. A logistic regression model was used to analyse the association between the genetic variants and OC risk or five year survival, adjusting for the first four principle components of all genotyped variants, the genotyping array used, and the age at recruitment for OC risk, or, the age at OC diagnosis for the five-year survival. All statistical tests were performed using GraphPad Prism^®^ (GraphPad Software, San Diego, CA, USA) and R software (http:///www.r-project.org/). Plot for the genetic associations were constructed through LocusZoom [19]. Values were considered as significant when *p* < 0.05.

## 3. Results

### 3.1. H2AX Is Over-Expressed in Ovarian Cancer and Predicts Survival

**Figure 1:**
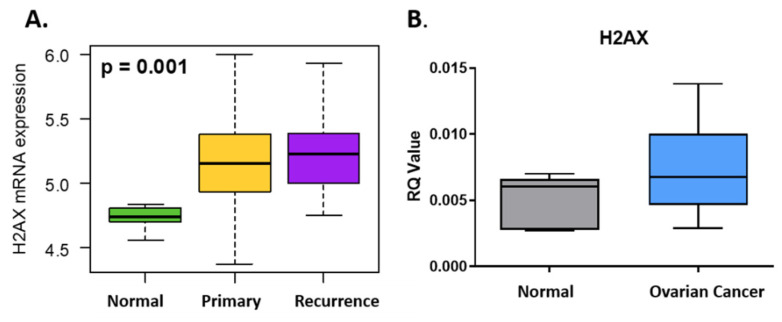
(**A**) *H2AX* is significantly (*p* = 0.001) overexpressed in OC tissues (Primary: *n* = 568, Recurrence: *n* = 17) against normal tissues (*n* = 8) in TCGA cohort. (**B**) *H2AX* is upregulated in stage III OC patients (*n* = 6) compared to controls (*n* = 3) quantified by qRT-PCR.

Expression of *H2AX* (or *H2AFX*) was significantly upregulated in the primary and recurrent ovarian cancer when compared to normal tissues in TCGA (Figure 1A). qRT-PCR in a small cohort of stage III ovarian cancer patients confirmed higher expression of *H2AX* when compared to age matched controls (Figure 1B).

**Figure 2:**
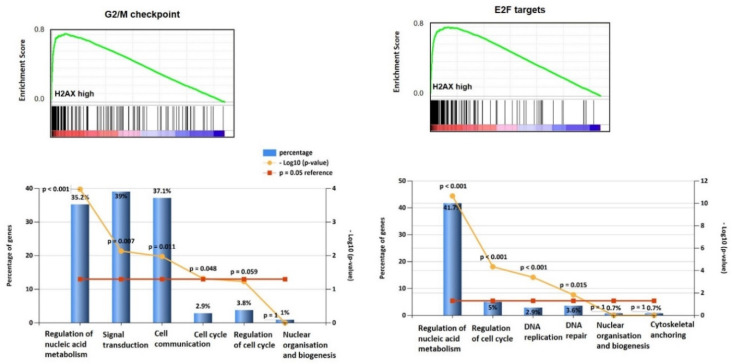
Gene enrichment on genes significantly associated with G2/M checkpoint, E2F targets, with significant biological processes of regulation of nucleic acid metabolism, signal transduction and cell communication for the G2/M group, and regulation of nuclei acid metabolism and cell cycle as well as DNA replication and repair for the E2F cohort.

**Figure 3:**
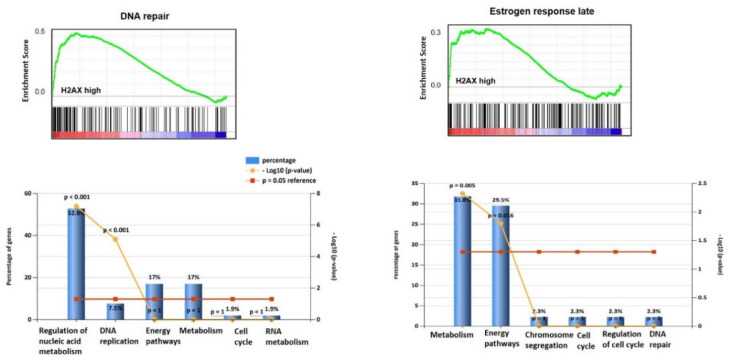
Gene enrichment on genes significantly associated with DNA repair and Estrogen response (late), with significant biological processes of regulation of nucleic acid metabolism and DNA replication for the DNA repair group, and metabolism and energy pathways for the Estrogen response cohort.

**Figure 4:**
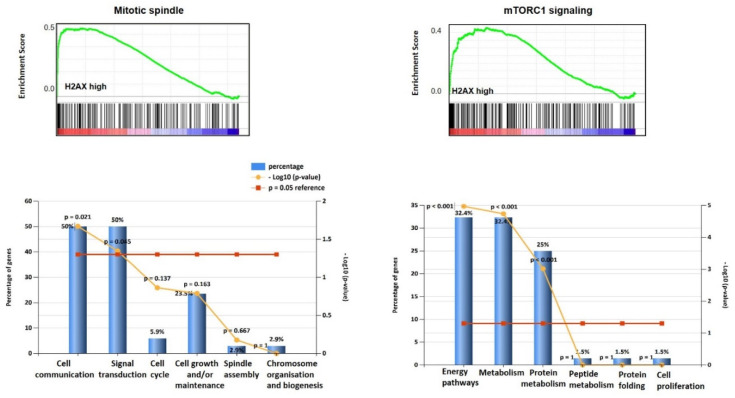
Gene enrichment on genes significantly associated with Mitotic spindle, and mTORC1 signalling, with significant biological processes of signal transduction and cell communication for the Mitotic spindle group, and energy pathways, metabolism and protein metabolism for the mTORC1 cohort.

Gene Set Enrichment Analysis (GSEA) demonstrated that gene sets (G2/M checkpoint: NES (normalized enrichment score) = 2.46, *p* < 0.001; E2F targets: NES = 2.30, *p* < 0.001 (Figure 2); DNA repair: NES = 2.15, *p* < 0.001; estrogen response: NES = 1.46, *p* = 0.048 (Figure 3); mitotic spindle: NES = 1.98, *p* < 0.001; mTORC1 signalling: NES = 1.6; *p* = 0.047 (Figure 4); and UV response: NES = 1.75, *p* = 0.004; were also enriched in the *H2AX* highly expressing ovarian cancers. In G2/M checkpoint, E2F target and DNA repair processes, genes associated with the regulation of nucleobase, nucleoside, nucleotide and nucleic acid metabolism were enriched.

Additionally, signal transduction and cell communication genes were enriched in the G2/M checkpoint set and cell cycle regulator and DNA replication/repair genes in the E2F gene set. From the estrogen response and mTORC1 signalling gene sets, metabolism (specifically protein metabolism for the mTORC1 set) and energy pathway genes were enriched. Finally, from the mitotic spindle set, genes from cell communication and signal transduction pathways were enriched.

**Figure 5:**
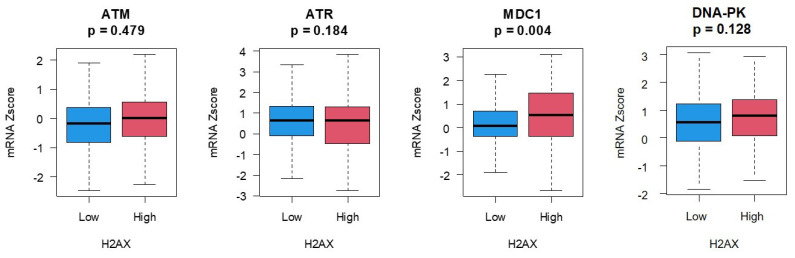
Differential expression of 4 key genes that alter the H2AX phosphorylation status in response to DSBs upon stratification of ovarian cancer patients to low- and high-expressing H2AX groups. Only Mediator of DNA Damage Checkpoint 1 (MDC1) was significantly upregulated in the high-*H2AX* expressing group.

We expanded on these observations by stratifying *H2AX* expression with expression of genes responsible for phosphorylating H2AX in response to DSBs, namely Ataxia telangiectasia mutated (ATM), Ataxia telangiectasia and Rad3 related (ATR), Mediator of DNA Damage Checkpoint 1 (MDC1) and DNA-dependent protein kinase (DNA-PK) (Figure 5). Of these four genes, only MDC1 was significantly upregulated in the high-*H2AX* expressing ovarian cancer group.

**Figure 6:**
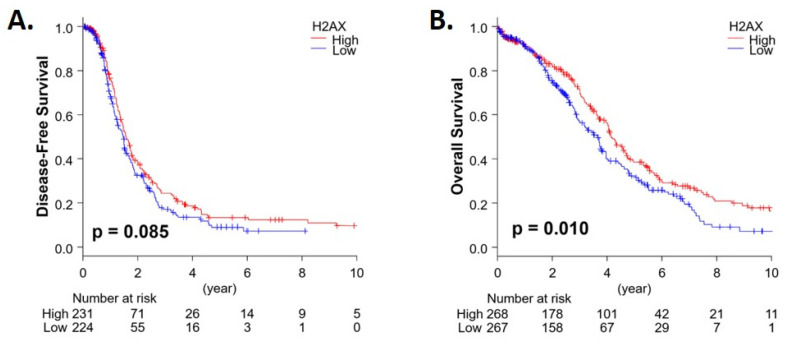
Kaplan–Meier (KM) plots for disease-free survival (**A**) and overall survival (**B**) in OC patients using the TCGA database.

In terms of prognostic value, higher expression of *H2AX* was associated with better overall survival (OS; *p* = 0.010), although there was no apparent difference in disease-free survival (DFS) in the TCGA OC cohort (Figure 6).

### 3.2. H2AX Protein Is Abundantly Expressed in Ovarian Cancer Tissues

**Figure 7:**
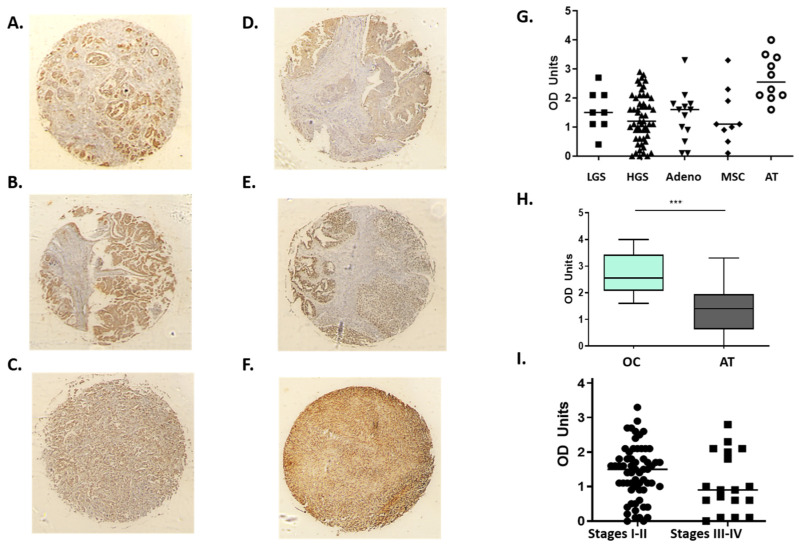
Staining of OC cores with H2AX. (**A**): Low grade serous (LGS), (**B**): High grade serous (HGS), (**C**): Mucinous adenocarcinoma (Adeno), (**D**): Metastatic serous carcinoma (MSC), (**E**): Endometrial adenocarcinoma, and (**F**): adjacent to tumour ovarian tissue (NAT). Abundance of staining was detected across all different OC types with an upregulation in NAT tissue (**G**). Significant protein upregulation of H2AX (*** *p* = 0.0003) when all OC cores were compared to NAT (**H**). Non-significant difference in H2AX staining between late (III and IV) and early stage (I and II) (**I**).

Following in silico analysis of *H2AX* in terms of concomitant gene expression and survival predictions, we explored the expression of H2AX protein in 100 cores of ovarian cancer patients and adjacent tissue (Figure 4). H2AX protein was abundantly expressed in both high- and low-grade serous samples, as well as in mucinous and endometrioid adenocarcinomas. Interestingly, normal adjacent control (NAT) ovarian tissue exhibited significantly higher expression of H2AX protein when compared to OC cores. Finally, a decrease in H2AX staining was noted in patients with late stage OC (stages III-IV, *n* = 18) when compared to early stages (I-II; *n* = 62; Figure 7).

### 3.3. Genetic Association Analysis for H2AX

**Figure 8:**
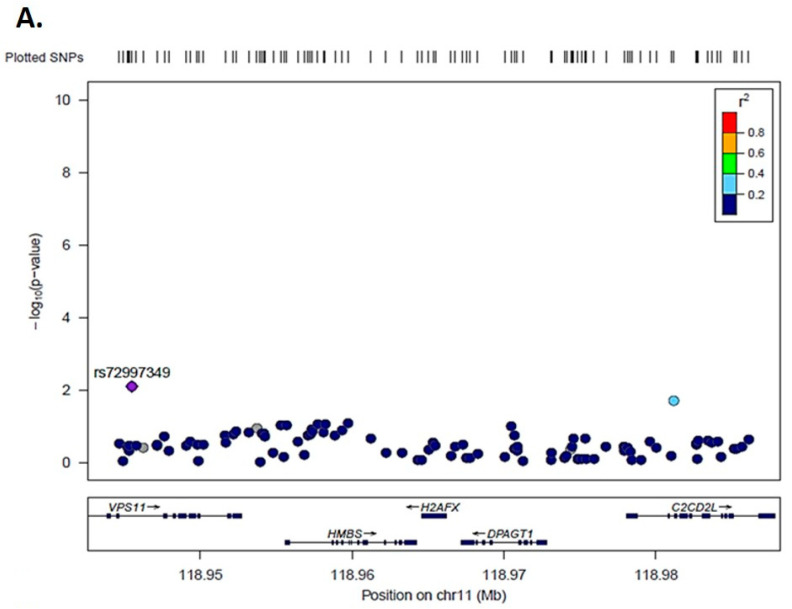

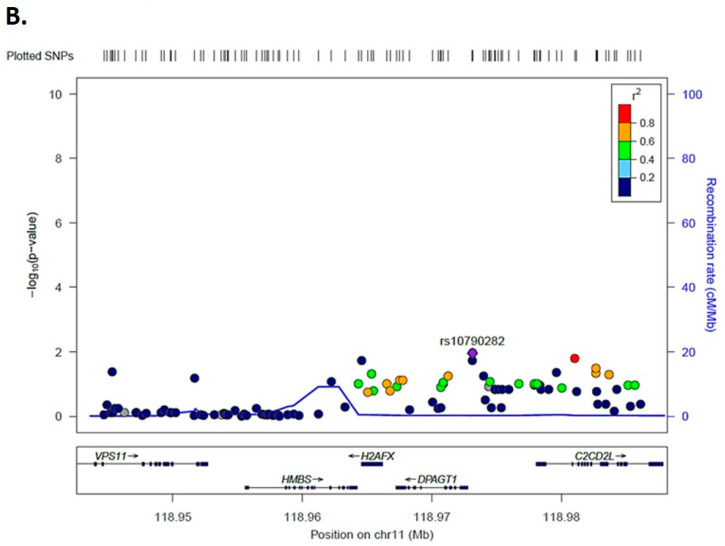
Polymorphism association analysis for *H2AX.* Reference SNP cluster rs72997349 (C-T) demonstrated a significant increase of risk for ovarian cancer (**A**), whereas Reference SNP cluster rs1079082 (A-G) demonstrated a significant increase in overall survival (**B**).

Using the 842 prevalent OC cases following QC, we identified one SNP associated with the outcome, rs72997349 (C-T) demonstrated a statistically significant increase of risk (*p* = 0.005) for OC (for the most common C-allele) with an odds ratio of 1.4 (95% CIs 1.1–1.8) (Figure 8A).

Using the 227 incident cases with 5-year follow up data, we identified 10 genetic variants in the area of the gene being associated with survival (Figure 8B). The top association in terms of lowest *p*-value was rs10790282 (A-G) with *p* = 0.011 and a protective effect of 0.58 OR for each G allele, or else an increasing OR of 1.72 per each A allele.

### 3.4. H2AX Expression Correlates with γ-H2AX Staining In Vitro

Here, we have examined whether transcriptional changes of the H2AX correlate with changes in γ-H2AX foci following induced DNA damage using H_2_O_2_ in *BRCA* wild-type and *BRCA2* mutant cell lines. For this, we used 4 OC cell lines as in vitro models: SKOV3 (*BRCA2* wild-type), PEO1 (*BRCA2*-mutant), PEO4 (*BRCA2*-silent mutant) and MDAH-2774 (*BRCA2* wild type). The number of γ-H2AX foci was measured using immunofluorescence. Prior to the experiment, we examined the number of γ-H2AX foci in *BRCA* wild-type and mutant OC cell lines to investigate whether *BRCA2* mutant status impacts HR deficiency and effective repair of DNA DSB in vitro.

**Figure 9:**
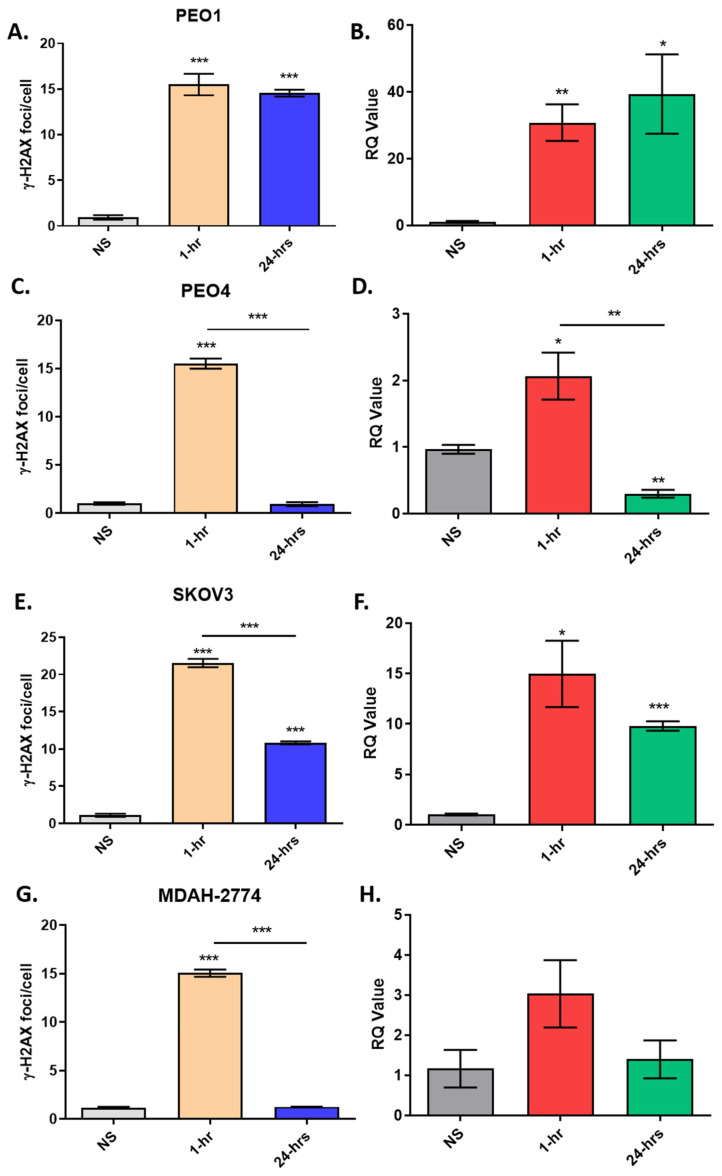
Treatment of cell lines with H_2_O_2_ and measurement of γ-H2AX foci (**A**,**C**,**E**,**G**) and *H2AX* gene expression (**B**,**D**,**F**,**H**) in BRCA2 mutant PEO1 cell line (**A**,**B**), in comparison with BRCA2 silent mutant cell line PEO4 (**C**,**D**) and BRCA2 wild type SKOV3 (**E**,**F**) and MDAH-2774 (**G**,**H**). NS; No Supplement. **Panel A**. 1-hr: *** *p* < 0.0001, 24-hrs: *** *p* < 0.0001 (compared to NS). **Panel B.** 1-hr: ** *p* = 0.0056, 24-hrs: * *p* = 0.0322 (compared to NS). **Panel C**. 1-hr: *** *p* < 0.0001 (compared to NS). *** *p* < 0.0001 (1-hr compared to 24-hrs). **Panel D.** 1-hr: * *p* = 0.0375, 24-hrs: ** *p* = 0.0016 (compared to NS). ** *p* = 0.0078 (1-hr compared to 24-hrs). **Panel E.** 1-hr: *** *p* < 0.0001, 24-hrs: *** *p* < 0.0001 (compared to NS). *** *p* < 0.0001 (1-hr compared to 24-hrs). **Panel F**. 1-hr: * *p* = 0.0134, 24-hrs: *** *p* < 0.0001 (compared to NS). **Panel G.** 1-hr: *** *p* < 0.0001 (compared to NS). *** *p* < 0.0001 (1-hr compared to 24-hrs).

We compared changes in gene expression to γ-H2AX staining in these cell lines after treatment with H_2_O_2_ to assess the correlation between mRNA expression and H2AX phosphorylation. It was shown that PEO1 (*BRCA2*-mutant) had a higher number of γ-H2AX foci in comparison to *BRCA*-wild type cell lines and especially PEO4 that a direct comparison can be drawn. The change of γ-H2AX in the cell lines is consistent with the alteration in *H2AX* gene expression (Figure 9; Supplementary Figures S1 and S2).

## 4. Discussion

In this study we provide a comprehensive overview of the changes at gene and protein level of H2AX in OC and suggest a possible predictive value as a biomarker. In silico analysis using TCGA database revealed that *H2AX* is overexpressed in OC when compared to controls. Interestingly, *H2AX* appears to be upregulated in most cancers when compared to controls with the exception of acute myeloid leukaemia (Supplementary Figure S3). It has been reported that *H2AX* expression level is significantly higher in triple negative breast cancers (TNBC) than non-TNBC and in HER2 positive breast cancers versus those without HER2 overexpression [20].

In terms of prognostic value, in TGCA OC cohort, we demonstrate that higher expression of *H2AX* is associated with better OS, but there is no apparent difference in disease free interval (DFI). However, a similar study using a qualitative protein expression scoring system for γ-H2AX reported a significantly reduced disease free interval amongst patients with high γ-H2AX expression, yet no significant correlation with OS in 63 OC patients [21]. Similarly, in renal cell and liver cancers, high expression of *H2AX* is an unfavourable prognostic marker (source: proteinatlas.org); advanced stage (I versus II–IV), *H2AX* high expressing breast tumours also showed significantly worse OS (*p* = 0.007) and DFS (*p* = 0.001).

Our gene set enrichment analysis, on the OC population with high H2AX expression, identified G2/M checkpoint, E2F targets, DNA repair, estrogen response, mitotic spindle and mTORC1 gene set associations. This fits well with the widely reported roles for H2AX as a key protein in DNA repair processes. We demonstrate that the OC population with high H2AX expression is associated with upregulation of several biological pathways, such as PI3K/AKT/mTOR, activated in ~50% serous OC [22]. This pathway, and primarily the mTORC1 complex, provides a balance between cellular resources such as amino acids and/or cellular stressors such as hypoxia to control cellular behaviour [23]. Emerging data also links mTOR with the aetiopathogenesis of OC. Our group has previously shown that inhibition of the mTOR pathway using rapamycin, and rapalogues induces a cytostatic effect on preclinical models of OC [24].

As expected, we demonstrate abundant expression of *H2AX* in high-grade serous OC, mucinous adenocarcinomas, and clear cell carcinomas. Surprisingly, we also show high expression in low-grade serous ovarian cancer. We also demonstrated no correlation between H2AX overexpression and FIGO stage, corroborating the study of 87 epithelial OC (EOC) patients, where γ-H2AX immunostaining was not significantly correlated with age, histopathological type, tumour differentiation, lymph node metastasis, FIGO stage or size of residual disease [21]. Low grade serous OC is generally categorised as Grade 1, only 8 of the 100 cores included in the microarray sere reported as G1/2 serous tumours. Historically, the ‘G2’ assigned serous cancers are now recognised a high-grade (G3) which could account for this discrepancy as the numbers of true G1 (low grade) cases were probably too small for comparison.

The most striking observation from the IHC study is that H2AX was significantly overexpressed in the normal adjacent tissue (NAT) when compared to the actual malignant area in all histopathological types of OC. This finding raises numerous questions as to how “normal” this tissue can be. Indeed, histologically normal tissue, routinely taken from the vicinity (<2 cm) of the malignant cells, is commonly used as a control in cancer studies. However, until recently, little was known about its transcriptomic profile, how it might be influenced by the malignant cells and how it might compare with healthy control tissue, either taken from some distance away (e.g., an unaffected ovary) or an age-matched healthy control. In a recent study using an array of cancers (including uterus and breast) it was shown that NAT tissue is distinct from both healthy and tumour tissues when they compared NAT, GTEX and TCGA transcriptomics from 8 cancer types [25]. NAT tissue undergoes extracellular matrix remodelling, wound healing-like processes, fibrosis, and an epithelial-to-mesenchymal transition (EMT) [26]. It seems likely that the upregulation we have seen in H2AX is related to crosstalk with the cancer microenvironment, ostensibly preconditioning the adjacent normal tissue. Another possibility for the differences between NAT, tumour and control tissues could be the potential involvement of microRNAs (miRNAs). For example, miR-24 upregulation in terminally differentiated blood cells, reduces H2AX [27]. There is therefore a possibility that this miRNA targets H2AX transcripts, since it appears to be present at ovarian level [28], based on the human miRNA tissue atlas. Of note, targeting miR-24 (along with miR-192-5p, miR-139-5p and miR-155-5p) could be a useful tool towards reducing or reversing cisplatin resistance [29].

We expanded on our observations by including data at population level, making use of the UK Biobank. We have identified two distinct clusters in the region of Chromosome 11q23.3 of OC patients that associate with increase in prevalence and poor prognosis. This is a chromosomal region that has been associated with loss of heterozygosity (LOH) in OC. For example, it has been shown that 11q22.3-q25 LOH has been associated with a more aggressive disease course in OC [30]. This was further corroborated by another study suggesting that LOH of 11q23.3-q24 (D11S1340 and D11S912), was also associated with an adverse disease course [31]. It should be noted that changes in this region are not entirely unique to OC.

Finally, γ-H2AX staining has been shown to be significantly higher in BRCA1/2 mutation-positive fallopian tube epithelium compared with control (BRCA wild-type) fallopian tube epithelium [32]. Here we demonstrate good correlation between the degree of phosphorylation of γ-H2AX foci with the gene expression of *H2AX*, in appropriate cell lines in vitro. We suggest that application of a DNA damaging agent such as H2O2 switches on cellular transcription machinery to generate H2AX proteins for phosphorylation to aid repair. These molecular events might be needed in order to provide a steady state of H2AX protein. It has been shown for example, that oncogenic transformation of human breast cancer epithelial cells drives a reduction in abundance of proteins [33]. Comparison of the BRCA2 mutant cell line (PEO4) compared to BRCA wild-type OC cell lines (MDA and SKOV3) and a cell-line with a silent BRCA2 mutation (PEO1) underpins the importance of BRCA2 in dsDNA repair. The intriguing rise in H2AX 24 h after H2O2 application in SKOV3 cells suggests that this line may exhibit a type of ‘BRCAness’. This refers to EOC (up to 50% of HGS) that exhibit a defective phenotype of homologous recombination repair (HRR) without BRCA mutations [34,35]. Of note, SKOV3 has 3 other genes mutated involved in HRR [36].

We acknowledge that our manuscript has certain limitations. There were only a small number of clinical samples for validation using qRT-PCR; we may have gained better insight by comparing γ-H2AX immunostaining with benign ovarian tissue from normal age-matched controls in addition to NAT tissue as well as perform γ-H2AX immunostaining on the same tissue microarray. The association study was informative but there is scope for expansion both in terms of number of cases and scope of analysis, studying prevalence for specific mutations for *H2AX*, as well looking into whether this gene is methylated in ovarian cancer. γ-H2AX immunostaining in EOC patients receiving chemotherapy would also be most informative to explore changes in *H2AX* expression following DNA damage in vivo. Finally, the impact of *BRCA1* mutations requires assessment in terms of *H2AX* expression. In conclusion, our study provides an insight into the expression and regulation of *H2AX* in OC. Further work is planned to identify whether it can be reliably used as a potential prognostic biomarker in the management of ovarian cancer.

## Supplementary Materials

The following are available online at https://www.mdpi.com/2077-0383/9/9/2844/s1, Figure S1: Detection of γ-H2AX foci in SKOV3 and MDAH274 cell lines, Figure S2: Detection of γ-H2AX foci in PEO1 and PEO2 cell lines; Figure S3: Pan-cancer gene expression of *H2AX,* Table S1: List of the tissues used for immunohistochemistry. TNM classification: tumor (T), nodes (N), and metastases (M); NAT: Normal Adjacent Tissue.
