# Supplementary material for: H2A Histone Family Member X (H2AX) Is Upregulated in Ovarian Cancer and Demonstrates Utility as a Prognostic Biomarker in Terms of Overall Survival
Source: J Clin Med. 2020 Sep 2;9(9):2844. doi: 10.3390/jcm9092844 (PMC7565050; doi:10.3390/jcm9092844)
Supplement: Supplementary file 1 [file jcm-09-02844-s001.pdf]

Supplementary Table S1. List of the tissues used for immunohistochemistry. TNM classification: tumor (T), nodes (N), and metastases (M); NAT: Normal Adjacent Tissue.

Position	No	Age	Sex	Organ/ Anatomic site	Pathology diagnosis	TNM	Grade	Stage	Type
A1	1	43	F	Ovary	Clear cell carcinoma	T1N0M0	-	I	Malignant
A2	2	61	F	Ovary	Clear cell carcinoma	T1aN0M0	-	IA	Malignant
A3	3	50	F	Ovary	Clear cell carcinoma	T1N0M0	-	I	Malignant
A4	4	40	F	Ovary	Clear cell carcinoma	T1N0M0	-	I	Malignant
A5	5	48	F	Ovary	Clear cell carcinoma (necrosis)	T2N0M0	*	II	Malignant
A6	6	50	F	Ovary	Low grade serous carcinoma	T2aN0M0	-	IIA	Malignant
A7	7	60	F	Ovary	Low grade serous carcinoma	T1cN0M0	-	IC	Malignant
A8	8	69	F	Ovary	Endometrioid adenocarcinoma	T1aN0M0	2	IA	Malignant
A9	9	41	F	Ovary	Low grade serous carcinoma	T1N0M0	-	I	Malignant
A10	10	37	F	Ovary	Low grade serous carcinoma	T1aN0M0	-	IA	Malignant
B1	11	25	F	Ovary	Low grade serous carcinoma	T1N0M0	-	I	Malignant
B2	12	34	F	Ovary	Low grade serous carcinoma	T1aN0M0	-	IA	Malignant
B3	13	59	F	Ovary	Low grade serous carcinoma	T1aN0M0	-	IA	Malignant
B4	14	34	F	Ovary	Low grade serous carcinoma	T1bN0M0	-	IB	Malignant
B5	15	56	F	Ovary	High grade serous carcinoma	T2N0M0	-	II	Malignant
B6	16	22	F	Ovary	High grade serous carcinoma	T2bN0M0	-	IIB	Malignant
B7	17	33	F	Ovary	High grade serous carcinoma	T1N0M0	-	I	Malignant

B8	18	56	F	Ovary	High grade serous carcinoma	T1N0M0	-	I	Malignant
B9	19	48	F	Ovary	High grade serous carcinoma	T1N0M0	-	I	Malignant
B10	20	43	F	Ovary	High grade serous carcinoma	T1N0M0	-	I	Malignant
C1	21	48	F	Ovary	High grade serous carcinoma	T2bN0M0	-	IIB	Malignant
C2	22	51	F	Ovary	High grade serous carcinoma	T3cN1M0	-	IIIC	Malignant
C3	23	42	F	Ovary	High grade serous carcinoma	T1N0M0	-	I	Malignant
C4	24	47	F	Ovary	High grade serous carcinoma	T3N0M0	-	III	Malignant
C5	25	64	F	Ovary	High grade serous carcinoma	T1N0M0	-	I	Malignant
C6	26	52	F	Ovary	High grade serous carcinoma	T1aN0M0	-	IA	Malignant
C7	27	53	F	Ovary	High grade serous carcinoma	T2N0M1	-	IV	Malignant
C8	28	60	F	Ovary	High grade serous carcinoma	T1bN0M0	-	IB	Malignant
C9	29	54	F	Ovary	High grade serous carcinoma	T3cN1M0	-	IIIC	Malignant
C10	30	53	F	Ovary	High grade serous carcinoma	T1N0M0	-	I	Malignant
D1	31	47	F	Ovary	High grade serous carcinoma	T1aN0M0	-	IA	Malignant
D2	32	48	F	Ovary	High grade serous carcinoma	T1N0M0	-	I	Malignant
D3	33	48	F	Ovary	High grade serous carcinoma	T3cN0M0	-	IIIC	Malignant
D4	34	53	F	Ovary	High grade serous carcinoma	T1cN0M0	-	IC	Malignant
D5	35	26	F	Ovary	High grade serous carcinoma	T3cN1M0	-	IIIC	Malignant

D6	36	35	F	Ovary	High grade serous carcinoma	T1aN0M0	-	IA	Malignant
D7	37	58	F	Ovary	High grade serous carcinoma	T1N0M0	-	I	Malignant
D8	38	60	F	Ovary	High grade serous carcinoma	T1aN0M0	-	IA	Malignant
D9	39	55	F	Ovary	High grade serous carcinoma	T1N0M0	-	I	Malignant
D10	40	67	F	Ovary	High grade serous carcinoma	T1aN0M0	-	IA	Malignant
E1	41	57	F	Ovary	High grade serous carcinoma	T3cN1M0	-	IIIC	Malignant
E2	42	41	F	Ovary	High grade serous carcinoma	T1N0M0	-	I	Malignant
E3	43	63	F	Ovary	High grade serous carcinoma with necrosis	T1N0M0	-	I	Malignant
E4	44	52	F	Ovary	High grade serous carcinoma	T2N0M0	-	II	Malignant
E5	45	66	F	Ovary	High grade serous carcinoma	T3N1M0	-	IIIC	Malignant
E6	46	52	F	Ovary	High grade serous carcinoma	T1cN0M0	-	IC	Malignant
E7	47	64	F	Ovary	High grade serous carcinoma	T3N1M0	-	IIIC	Malignant
E8	48	62	F	Ovary	High grade serous carcinoma	T2N0M0	-	II	Malignant
E9	49	42	F	Ovary	High grade serous carcinoma	T2N0M0	-	II	Malignant
E10	50	49	F	Ovary	High grade serous carcinoma	T2N0M0	-	II	Malignant
F1	51	59	F	Ovary	High grade serous carcinoma with necrosis	T1cN0M0	-	IC	Malignant
F2	52	42	F	Ovary	High grade serous carcinoma (sparse)	T3cN1M0	-	IIIC	Malignant

F3	53	49	F	Ovary	High grade serous carcinoma	T1aN0M0	-	IA	Malignant
F4	54	69	F	Ovary	High grade serous carcinoma	T2N0M0	-	II	Malignant
F5	55	42	F	Ovary	High grade serous carcinoma	T3N1M0	-	IIIC	Malignant
F6	56	53	F	Ovary	High grade serous carcinoma with necrosis	T1aN0M0	-	IA	Malignant
F7	57	47	F	Ovary	High grade serous carcinoma	T1cN0M0	-	IC	Malignant
F8	58	49	F	Ovary	High grade serous carcinoma	T3N1M0	-	IIIC	Malignant
F9	59	52	F	Ovary	High grade serous carcinoma	T3cN1M0	-	IIIC	Malignant
F10	60	55	F	Ovary	High grade serous carcinoma	T3N1M0	-	IIIC	Malignant
G1	61	52	F	Ovary	High grade serous carcinoma	T2N0M0	-	II	Malignant
G2	62	51	F	Ovary	High grade serous carcinoma	T2N0M0	-	II	Malignant
G3	63	41	F	Ovary	High grade serous carcinoma	T1aN0M0	-	IA	Malignant
G4	64	56	F	Ovary	High grade serous carcinoma	T3N0M0	-	III	Malignant
G5	65	55	F	Ovary	High grade serous carcinoma	T1N0M0	-	I	Malignant
G6	66	48	F	Ovary	High grade serous carcinoma	T3aN0M0	-	IIIA	Malignant
G7	67	60	F	Ovary	High grade serous carcinoma	T2bN0M0	-	IIB	Malignant
G8	68	51	F	Ovary	High grade serous carcinoma	T1aN0M0	-	IA	Malignant
G9	69	46	F	Ovary	Mucinous papillary adenocarcinoma (necrosis)	T1aN0M0	*	IA	Malignant
G10	70	49	F	Ovary	Endometrioid	T1N0M0	2	I	Malignant

					adenocarcinoma				
H1	71	34	F	Ovary	Mucinous adenocarcinoma	T1bN0M0	2	IB	Malignant
H2	72	37	F	Ovary	Mucinous adenocarcinoma	T1aN0M0	2	IA	Malignant
H3	73	39	F	Ovary	Mucinous adenocarcinoma with necrosis	T1aN0M0	2	IA	Malignant
H4	74	54	F	Ovary	Mucinous adenocarcinoma	T2aN0M0	2	IIA	Malignant
H5	75	41	F	Ovary	Mucinous adenocarcinoma with necrosis	T1bN0M0	2--3	IB	Malignant
H6	76	50	F	Ovary	Mucinous adenocarcinoma	T3cN1M0	3	IIIC	Malignant
H7	77	52	F	Ovary	Mucinous adenocarcinoma	T1bN0M0	3	IB	Malignant
H8	78	38	F	Ovary	Mucinous adenocarcinoma	T1N0M0	3	I	Malignant
H9	79	29	F	Ovary	Mucinous adenocarcinoma	T3N0M0	3	III	Malignant
H10	80	58	F	Ovary	Endometrioid adenocarcinoma	T1aN0M0	3	IA	Malignant
I1	81	47	F	Lymph node	Metastatic serous carcinoma from ovary	-	-	-	Metastasis
I2	82	48	F	Lymph node	Metastatic serous carcinoma from ovary	-	-	-	Metastasis
I3	83	46	F	Lymph node	Metastatic serous carcinoma from ovary		-	-	Metastasis
I4	84	54	F	Lymph node	Metastatic serous carcinoma from ovary	-	-	-	Metastasis
I5	85	83	F	Lymph node	Metastatic serous carcinoma from ovary	-	*	-	Metastasis

I6	86	48	F	Lymph node	Metastatic clear cell carcinoma from ovary	-	-	-	Metastasis
I7	87	56	F	Pelvic cavity	Metastatic serous carcinoma of fibrofatty tissue from ovary of No.64	-	-	-	Metastasis
I8	88	57	F	Greater omentum	Metastatic serous carcinoma from ovary	-	-	-	Metastasis
I9	89	50	F	Lymph node	Metastatic serous carcinoma from ovary	-	-	-	Metastasis
I10	90	53	F	Pelvic cavity	Metastatic serous carcinoma of fibrofatty tissue from ovary	-	-	-	Metastasis
J1	91	69	F	Ovary	Adjacent normal ovary tissue	-	-	-	NAT
J2	92	48	F	Ovary	Adjacent normal ovary tissue	-	-	-	NAT
J3	93	53	F	Ovary	Adjacent normal ovary tissue	-	-	-	NAT
J4	94	42	F	Ovary	Adjacent normal ovary tissue	-	-	-	NAT
J5	95	42	F	Ovary	Adjacent normal ovary tissue	-	-	-	NAT
J6	96	40	F	Ovary	Adjacent normal ovary tissue	-	-	-	NAT
J7	97	59	F	Ovary	Adjacent normal ovary tissue	-	-	-	NAT
J8	98	42	F	Ovary	Adjacent normal ovary tissue	-	-	-	NAT
J9	99	35	F	Ovary	Adjacent normal ovary tissue	-	-	-	NAT
J10	100	45	F	Ovary	Adjacent normal ovary tissue	-	-	-	NAT

-	0	42	M	Adrenal gland	Pheochromocytoma (tissue marker)	-	Malignant
